# Associations between body mass index and all-cause mortality among individuals with psoriasis: results from the NHANES database retrospective cohort study

**DOI:** 10.3389/fnut.2024.1407454

**Published:** 2024-06-05

**Authors:** ZhiHong Wei, GuanHua Nie, Christian D. Sadik, Dan Shan

**Affiliations:** ^1^Department of Dermatology, Allergy, and Venereology, University of Lübeck, Lübeck, Germany; ^2^School of Medicine, National University of Ireland, Galway, Ireland; ^3^Institute for Experimental and Clinical Pharmacology and Toxicology, Center of Brain, Behavior and Metabolism, University of Lübeck, Lübeck, Germany; ^4^Faculty of Health and Medicine, Lancaster University, Lancaster, United Kingdom

**Keywords:** psoriasis, body mass index, BMI, all-cause mortality, obesity, NHANES, overweight

## Abstract

**Background:**

Previous findings imply a potential positive association between BMI and all-cause mortality in individuals with psoriasis, yet direct evidence remains absent. This study aimed to fill this gap.

**Methods:**

We utilized data from the National Health and Nutrition Examination Survey (NHANES) for the periods 2003–2006 and 2009–2014. Participants’ BMI was categorized as lean (<25), overweight (25 ≤ BMI < 30), and obese (BMI ≥ 30). Psoriasis status was determined through self-reporting. The main outcome measured was all-cause mortality up to December 2019. We accounted for multiple covariates, such as sociodemographic factors and histories of smoking and alcohol consumption. Our statistical analyses mainly included Kaplan–Meier survival analysis, Restricted Cubic Spline (RCS) and Multivariate Cox Regression (MCR). We also applied propensity score matching (PSM) to verify the robustness of our findings.

**Results:**

Among 22,876 participants, 618 (2.70%) reported a history of psoriasis. An overall effect from the MCR analysis showed that, among individuals with psoriasis, a higher baseline BMI was independently associated with an increased risk of all-cause mortality, noting a 5.5% rise in mortality risk per BMI unit [hazard ratio (HR) = 1.055, 95% CI: 1.004–1.110, *p* = 0.035]. This significant relationship persisted after PSM. A statistically significant positive correlation was consistent among males, smokers, and individuals younger than 60. However, no such association was found in individuals without a history of psoriasis. Additionally, no significant difference in mortality risk was found between lean and overweight groups with psoriasis, according to the RCS regression and stratified analysis.

**Conclusion:**

Our findings indicated a trend that, higher BMIs significantly correlated with increased risks of all-cause mortality in people with psoriasis, particularly among obese ones. However, the impact of being overweight on this relationship remains underexplored. Moreover, the necessity to employ alternative metrics beyond BMI for body fat assessment to further investigate these associations is critical.

## 1 Introduction

Psoriasis, a chronic and immune-mediated inflammatory skin disorder with no cure, is characterized by red, scaly plaques. These plaques most frequently appear on the elbows, knees, scalp, and lower back, but can affect any skin surface (1). It impacts millions worldwide, with a notably higher prevalence in Western countries characterized by older populations and higher income levels (2). The rate of psoriasis varies significantly by region, from 0.14% in East Asia to 1.99% in Australasia, with also substantial prevalence in Western Europe (1.92%), Central Europe (1.83%), and North America (1.50%) (1). Considered now as a serious non-communicable systemic disease, psoriasis is linked with a wide range of co-morbidities, including psychological, metabolic, arthritic, and cardiovascular issues (1). Prior research has indicated that psoriasis can be independently linked to a higher risk of mortality (3), presenting complex challenges for both dermatological and systemic health care, especially in individuals with more severe forms of the disease (1, 2).

The relationship between psoriasis and obesity has been a subject of increasing interest within the medical research community (4–6), given the growing evidence suggesting that obesity, as a prevalent and potentially modifiable risk factor, can exacerbate the clinical trajectory of psoriasis and vice versa (4, 6, 7). It was found that the inflammatory pathways shared between obesity and psoriasis, which involve cytokines such as TNF-α and IL-6, may suggest a biological plausibility for their bidirectional correlations (8–10). Both conditions are associated with a heightened risk of mortality (3, 11).

Body mass index (BMI) serves as a convenient and reliable approach for evaluating obesity in most people most of the time (12). Aune et al. identified a dose–response relationship between BMI and the risk of psoriasis onset, noting the lowest risk at a BMI of 20 and a significant risk increase starting from a BMI of 22.5–24 (13, 14). However, the association between BMI and mortality remains elusive. A US retrospective cohort study, spanning recent two decades, revealed a U-shaped relationship relating weight to mortality, indicating highest death rates at two extreme sides of BMI. Interestingly, being overweight (BMI 25–30) did not correlate with increased mortality (15). Contrarily, a meta-analysis suggested that both overweight and obesity are linked to a higher risk of all-cause mortality (16). Tobias et al. provided additional evidence to support a strong association between escalating BMI and increased mortality starting from a BMI of 25 kg/m^2^ (17). Despite the well-documented relationship between psoriasis, obesity, and increased risks of morbidity and mortality, the role of BMI, especially in the categories considered underweight or overweight, in affecting mortality is still unclear. The mediating effects of factors such as smoking, income and educational level can further complicate this association (18, 19).

A study suggested that higher BMIs are associated with more severe psoriasis among individuals who are lean or overweight (20). Another study showed that more severe psoriasis could be related to an increased risk of mortality (21). Based on these prior findings, one may speculate that, among non-obese individuals with psoriasis, a lower BMI can be associated with a reduced psoriasis severity, and thus a reduced risk of mortality. However, no current study provides direct evidence about this. Additionally, the influence of potentially confounding factors such as sex, age, previous smoking history, and comorbidities on such relationship is uncertain.

The National Health and Nutrition Examination Survey (NHANES) can provide invaluable resource for studying these associations, based on a large, nationally representative sample of the U.S. population. Therefore, through analyzing its data, we aimed to provide evidence about the impact of BMI on the risk of all-cause mortality in individuals with a history of psoriasis. We also extended our investigation to ascertain if this link persists in those without psoriasis. In addition, we aimed scrutinize the influence of various factors, such as age, sex, prior smoking history, on this critical association, aiming to inform clinical management and intervention strategies effectively.

## 2 Materials and methods

### 2.1 Study design and population

The NHANES database, an ongoing program conducted by the National Center for Health Statistics (NCHS) of the Centers for Disease Control and Prevention (CDC), offers information into the nutritional and health statuses of adults and children in the U.S. through population estimates. This survey employs a stratified, multistage probability design for selecting a representative sample of the U.S. population, gathering data through personal structured interviews at participants’ homes, health examinations at mobile examination centers, and laboratory analyses of specimens (22). The protocols for NHANES received approval from the Research Ethics Review Board at NCHS (23). Informed written consent was obtained from all participants involved in NHANES. The datasets produced and analyzed in this study can be accessed on the NHANES website.^1^

In the current study, we downloaded and analyzed the continuous NHANES data from 2003–2006 and 2009–2014, which encompass extensive details, including BMI values and the presence of psoriasis. The 2007–2008 NHANES cycle was not included due to the lack of psoriasis-related data.

### 2.2 Assessment of BMI and psoriasis

Participants’ BMI baseline data were determined by the formula: weight/height^2^ (kg/m^2^). The height and weight of participants were measured by professional surveyors. They were categorized into three groups based on their BMI: a lean group (BMI < 25), an overweight group (25 ≤ BMI < 30), and an obese group (BMI ≥ 30) (24). Follow-up data regarding BMI among these participants were not available.

All participants provided self-reported information on their dermatological conditions related to psoriasis. History of psoriasis was identified though a positive response to the question, “Have you ever been told by a health care provider that you have psoriasis?” The severity of psoriasis was assessed based on responses to the question, “What is the current severity of your psoriasis?” Participants could indicate their condition as (1). little or no psoriasis; (2). only a few patches; (3). scattered patches; or (4). extensive psoriasis.

### 2.3 Ascertainment of mortality

This study’s main outcome was all-cause mortality, which includes all deaths occurring during the follow-up period extending through December 2019. Confirmation of deaths was obtained through linkage with the National Death Index records (25).

### 2.4 Inclusion of covariates

We extracted the data pertaining participants’ sociodemographic details, as well as their histories of smoking and alcohol use, the severity of their current psoriasis, and the presence of diabetes, hypertension, and dyslipidemia. Individuals who had smoked fewer than 100 cigarettes in their lifetime were categorized as non-smokers, whereas those who had smoked 100 or more cigarettes were classified as smokers (22). Alcohol consumers were identified in participants who reported consuming at least 12 alcoholic drinks in their lifetime (26). We subjectively categorized the severity of current psoriasis among participants, including none (i.e., indicating little or no psoriasis), mild (i.e., indicating only a few patches), moderate (i.e., indicating scattered patches), and severe (i.e., indicating extensive psoriasis). Diabetes was determined by a glycosylated hemoglobin level of 6.5% or higher, a self-reported physician diagnosis, or the use of insulin (27). Hypertension was identified by a blood pressure reading of 140/90 mmHg or higher, a self-reported physician diagnosis, or the use of medication for hypertension (28). Dyslipidemia was defined by a serum total cholesterol level greater than 6.2 mmol/L or a self-reported physician diagnosis of dyslipidemia (29).

### 2.5 Statistical analysis

Continuous variables were reported as mean ± SD (standard deviation) or median and interquartile ranges, while categorical variables were presented as percentages. To compare continuous variables, we utilized the t-test or Mann–Whitney U test as appropriate. Categorical variables were compared using the chi-square test or Fisher’s exact test. Kaplan–Meier method was used to analyze survival probability of psoriatic individuals over time, based on BMI category. Restricted cubic spline (RCS) regression was employed to investigate the non-linear association between BMI (as a continuous variable) and all-cause mortality risk among psoriatic individuals. Multivariate Cox proportional hazards regression was also employed, in order to estimate hazard ratios (HRs) for all-cause mortality in relation to BMI in individuals with or without psoriasis (the latter one serves as an extra analysis), as well as for psoriatic severity based on BMI among psoriatic individuals. Stratified analyses were performed based on sex (men or women), age (≤60 or > 60 years old), and previous smoking status to explore their potential modifying effects, which were assessed by testing multiplicative interaction terms. Fisher’s exact test was employed to analyze the differing mortality rates among individuals with mild psoriasis and those with moderate-to-severe psoriasis based on BMI category groups.

In multivariate Cox regression models, we developed two statistical models for analyses. Model 1 adjusted for age (as a continuous variable), sex (male or female), and race/ethnicity (non-Hispanic White or other). Model 2 included adjustments for age, sex, race/ethnicity, education level (less than high school, high school or equivalent, or college or above), family income-to-poverty ratio (PIR) (as a continuous variable), previous alcohol consumption, previous smoking status, and the presence of diabetes, hypertension, dyslipidemia, and current severity of psoriasis (mild or moderate-to-severe).

Sensitivity analyses including several inclusion criteria for psoriatic participants were conducted to assess the stability of our results. Firstly, to mitigate potential biases arising from underlying confounding factors that may accelerate the dying process of psoriatic participants, those who died within the first 2 years of follow-up were excluded (*n* = 12). Secondly, we used the propensity score matching (PSM) approach to balance baseline characteristics between obese (BMI ≥ 30) and non-obese (BMI < 30) groups at a 1:2 ratio. The predefined factors employed for these adjustments included: age, sex, race/ethnicity, educational level, PIR, previous alcohol consumption, previous smoking status, the presence of hypertension, diabetes and dyslipidemia, and the current severity of psoriasis. Finally, we conducted a third sensitivity analysis based on the combined inclusion criteria from the previous two analyses.

All statistical analyses were conducted using IBM SPSS Statistics 27 and R software version 4.3.1. A two-sided *p*-value of <0.05 was deemed to indicate statistical significance.

## 3 Results

**Figure 1:**
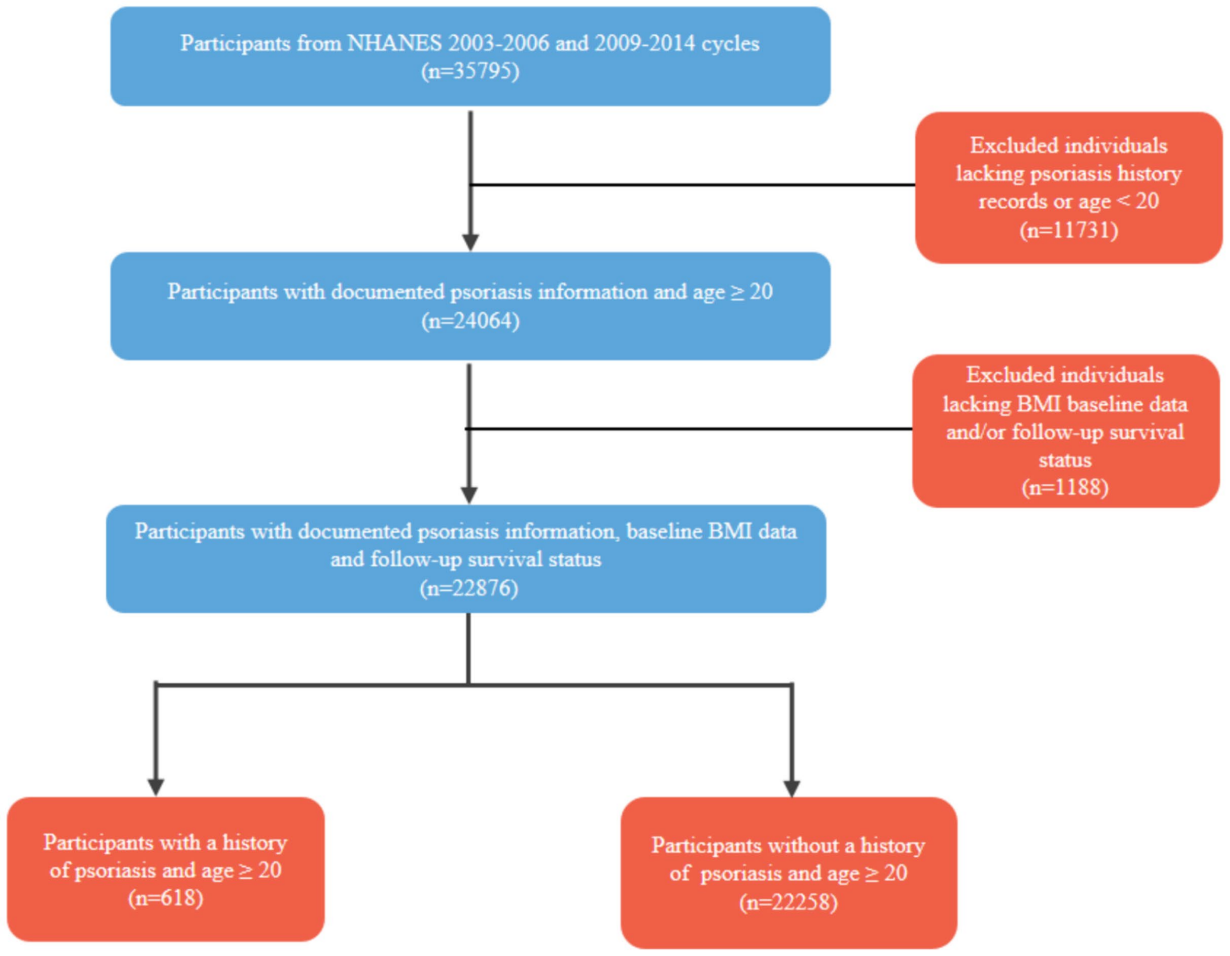
Flow-chart of participant selection process. BMI, body mass index; NHANES, National Health and Nutrition Examination Survey.

A total of 22,876 individuals, with or without a history of psoriasis, aged 20 years old and above were included in the final analysis. 618 (2.7%) were identified as having a history of psoriasis (see Figure 1 for participant selection details). The lowest recorded baseline BMI among all participants was 16.56, and the highest was 63. Among individuals with histories of psoriasis, only 3 participants had a baseline BMI below 18.5, and 26 participants had a baseline BMI exceeding 45.

### 3.1 Baseline characteristics

Compared to those without histories of psoriasis, individuals with histories of the condition were generally older, predominantly non-Hispanic White, had higher BMI levels, and were more likely to smoke (refer to Supplementary Table S1, all *p*-values <0.05). The distribution of these characteristics among participants with psoriasis, based on BMI categories, was detailed in Table 1. Notably, the overweight group (25 ≤ BMI < 30) had the highest percentages of male participants, non-Hispanic Whites, and individuals with the highest Poverty Income Ratio (PIR), with significant differences observed (*p* < 0.05). However, no significant differences were noted in other characteristics across BMI groups (*p* > 0.05). Furthermore, Supplementary Tables S2, S3 display the baseline characteristics of individuals with psoriasis, categorized by obesity status, after sole Propensity Score Matching (PSM), and after PSM combined with the exclusion of participants who died within the first two years of follow-up. In these PSM data, no statistically significant differences were found between obese and non-obese groups with histories of psoriasis. In addition, Supplementary Table S4 shows that, in the psoriatic individuals, among various causes of death, the incidence of cardiovascular-related death events was significantly higher in the obese group compared to the lean and overweight groups (*p* = 0.005).

**Table 1:** Baseline characteristics of psoriatic individuals according to BMI.

	BMI < 25 (*n* = 142)	25 ≤ BMI < 30 (*n* = 211)	BMI ≥ 30 (*n* = 265)	*p*-value
Age, years old	46.55 ± 17.35	50.62 ± 16.68	49.75 ± 15.14	0.058
Male, n%	57 (40.10)	117 (55.50)	115 (43.40)	0.006
Race/Ethnicity				<0.001
Mexican American	7 (4.93)	11 (5.21)	33 (12.5)	
Other Hispanic	8 (5.63)	17 (8.06)	23 (8.68)	
Non-Hispanic White	90 (63.38)	134 (63.51)	151 (56.98)	
Non-Hispanic Black	11 (7.75)	27 (12.80)	45 (16.98)	
Other race	26 (18.31)	22 (10.42)	13 (4.86)	
Educational attainment				0.173
Less than 9th grade	9 (6.34)	14 (6.64)	18 (6.79)	
9-11th grade	17 (11.97)	31 (14.69)	34 (12.83)	
High school	33 (23.24)	44 (20.85)	62 (23.40)	
Some college or AA degree	41 (28.87)	58 (27.49)	99 (37.36)	
College graduate or above	42 (29.58)	64 (30.33)	52 (19.62)	
PIR	2.54 ± 1.68	2.86 ± 1.77	2.43 ± 1.64	0.025
Smoking	80 (56.34)	113 (53.55)	151 (56.98)	0.743
Drinking	54 (38.03)	61 (28.90)	104 (39.25)	0.060
Hypertension	36 (25.35)	42 (19.91)	58 (21.89)	0.835
Diabetes	6 (4.23)	19 (9.00)	15 (5.67)	0.456
Dyslipidemia	29 (20.42)	49 (23.22)	53 (20.00)	0.391
Severity of psoriasis				0.874
Mild	115 (80.99)	176 (83.41)	208 (78.49)	
Moderate-to-severe	27 (19.01)	35 (16.59)	57 (21.51)	

AA, the associate in arts degree. PIR, poverty income ratio.

### 3.2 Stratified analysis of all-cause mortality

The initial analysis explored the impact of BMI categories (i.e., lean, overweight, and obese) on all-cause mortality among those with a history of psoriasis. Out of 2,098 deceased participants over a median follow-up of 8.9 years (IQR = 6.4), 81 (3.9%) had psoriasis, and 2,017 (96.1%) did not.

After adjusting for multiple covariates, the stratified analysis revealed a significantly higher all-cause mortality in the obese group (BMI ≥ 30) (HR: 4.239, 95% CI: 1.442–12.458, *p* = 0.020), but not in the overweight group (HR: 0.238, 95% CI: 0.052–1.092), compared to the lean group (see Table 2). The obese group was also significantly associated with higher mortality risks in the subgroups of men, women, age ≤ 60, age > 60, smokers, non-smokers, individuals with mild psoriasis, and individuals with moderate-to-severe psoriasis. However, insignificant differences were observed in all these subgroups between the lean and overweight groups. Additionally, no significant interactions were observed in the stratified analyses (all *p* > 0.05).

**Table 2:** Stratified analysis of the risk of all-cause mortality based on BMI category of psoriatic individuals.

	BMI < 25 (lean)	25 ≤ BMI < 30 (overweight)	BMI ≥ 30 (obese)	*p* value	P_interaction_
Total					
No. deaths/total	12/142	23/211	46/265	0.020	
Crude	1	0.767 (0.473–1.244)	1.783 (1.149–2.767)		
Model 1	1	0.649 (0.396–1.062)	2.101 (1.339–3.297)		
Model 2	1	0.238 (0.052–1.092)	4.239 (1.442–12.458)		
Sex					0.762
Men					
No. deaths/total	5/57	13/117	26/115	0.016	
Crude	1	1.960 (0.772–4.973)	2.235 (1.225–4.079)		
Model 1	1	2.235 (0.874–5.710)	3.036 (1.631–5.652)		
Model 2	1	1.892 (0.329–9.121)	3.737 (1.660–8.415)		
Women					
No. deaths/total	7/85	10/94	20/150	0.482	
Crude	1	1.546 (0.679–3.520)	1.432 (0.750–2.734)		
Model 1	1	1.400 (0.613–3.197)	1.388 (0.724–2.661)		
Model 2	1	0.666 (0.109–4.064)	4.116 (1.886–19.172)		
Age, years					0.585
Age ≤ 60					
No. deaths/total	4/112	6/148	20/199	0.028	
Crude	1	0.527 (0.216–1.290)	2.631 (1.231–5.621)		
Model 1	1	0.527 (0.215–1.292)	2.238 (1.046–4.786)		
Model 2	1	0.397 (0.099–1.581)	2.440 (1.030–5.820)		
Age > 60					
No. deaths/total	8/30	17/63	26/66	0.250	
Crude	1	0.725 (0.405–1.297)	1.561 (0.901–2.704)		
Model 1	1	0.671 (0.369–1.222)	2.212 (1.233–3.970)		
Model 2	1	0.127 (0.010–1.123)	2.250 (1.210–4.200)		
Previous smoking status					0.668
Smoking					
No. deaths/total	8/80	19/113	37/151	0.022	
Crude	1	0.880 (0.515–1.505)	1.825 (1.111–2.998)		
Model 1	1	0.785 (0.457–1.349)	2.009 (1.212–3.332)		
Model 2	1	0.742 (0.310–1.751)	2.040 (1.240–3.370)		
Non-smoking					
No. deaths/total	4/62	4/98	9/114	0.516	
Crude	1	0.547 (0.178–1.677)	1.568 (0.605–4.066)		
Model 1	1	0.258 (0.078–0.852)	2.728 (1.002–7.426)		
Model 2	1	0.049 (0.002–1.042)	5.580 (2.110–14.78)		
Severity of psoriasis					0.112
Mild					
No. deaths/total	12/115	17/176	32/208	0.148	
Crude	1	0.544 (0.246–1.201)	1.855 (0.943–3.651)		
Model 1	1	0.496 (0.224–1.101)	0.785 (0.400–1.542)		
Model 2	1	0.334 (0.111–1.007)	3.218 (1.316–7.871)		
Moderate-to-severe					
No. deaths/total	0/27	6/35	11/57	0.089	
Crude	1	0.822 (0.218–3.099)	3.420 (0.907–12.898)		
Model 1	1	0.852 (0.224–3.238)	3.474 (0.900–13.401)		
Model 2	1	0.225 (0.008–6.429)	1.287 (1.006–14.587)		

Data were presented as HR (95% CI) unless indicated otherwise. Model 1 was adjusted for age (continuous), sex (male or female), race/ethnicity (non-Hispanic White or other). Model 2 was adjusted for age (continuous), sex (male or female), race/ethnicity, education level, family income-to-poverty ratio, previous drinking and smoking history, and presence of diabetes, hypertension, dyslipidemia, and current psoriatic severity, where appropriate.

### 3.3 Incidence rate of all-cause mortality by BMI group among psoriatic individuals

The Kaplan–Meier survival analysis curves suggested significant differences in mortality rates across BMI groups among psoriatic individuals by the end of the follow-up period (Lean: 8.4% vs. Overweight: 10.9% vs. Obese: 17.4%, log-rank *p* = 0.026, see Supplementary Figure S1A). This pattern was also significant among participants aged ≤60 (Lean: 3.5% vs. Overweight: 4.1% vs. Obese: 16.8%, log-rank *p* = 0.033, see Supplementary Figure S1C), and those considered as smokers (Lean: 10.0% vs. Overweight: 16.8% vs. Obese: 24.5%, log-rank *p* = 0.024, see Supplementary Figure S1D), but not among those aged >60 (log-rank *p* = 0.276, see Supplementary Figure S1B) or those considered as non-smokers (log-rank *p* = 0.534, see Supplementary Figure S1E).

### 3.4 Association between BMI and the risk of all-cause mortality among participants with or without psoriasis

Multivariate Cox regression analyses indicated that a higher BMI was associated with an increased risk of all-cause mortality in individuals with a history of psoriasis, even after adjusting for age, sex, race/ethnicity, education level, family income-to-poverty ratio, previous alcohol consumption, previous smoking status, the presence of diabetes, hypertension, and dyslipidemia, and current psoriatic severity. The risk of death increased by 5.5% per BMI unit increase (HR: 1.055, 95% CI: 1.004–1.110, *p* = 0.035, see Table 3). However, the association between a higher BMI value and an increased all-cause mortality risk was not significantly observed in those without a history of psoriasis (Supplementary Table S5, *p* = 0.553).

**Table 3:** Multivariate Cox Regression for all-cause mortality in patients with psoriasis.

		HR	95% CI	*p* value
BMI				
	Crude	1.043	1.017–1.071	0.003
	Model 1	1.049	1.021–1.077	<0.001
	Model 2	1.055	1.004–1.110	0.035
Men				
BMI	Crude	1.075	1.031–1.120	<0.001
	Model 1	1.095	1.049–1.144	<0.001
	Model 2	1.096	1.014–1.186	0.021
Women				
BMI	Crude	1.030	0.994–1.067	0.107
	Model 1	1.022	1.054–1.108	0.226
	Model 2	1.047	0.985–1.113	0.139
Age ≤ 60				
BMI	Crude	1.066	1.025–1.108	0.002
	Model 1	1.045	1.005–1.087	0.027
	Model 2	1.275	1.051–1.540	0.011
Age > 60				
BMI	Crude	1.023	0.987–1.061	0.215
	Model 1	1.053	1.013–1.094	0.009
	Model 2	1.080	1.019–1.145	0.007
Smoking				
BMI	Crude	1.042	1.014–1.070	0.003
	Model 1	1.042	1.013–1.071	0.004
	Model 2	1.041	1.005–1.077	0.026
Non-smoking				
BMI	Crude	1.032	0.965–1.103	0.362
	Model 1	1.055	0.979–1.137	0.160
	Model 2	1.034	0.893–1.203	0.662

Model 1 was adjusted for age (continuous), sex (male or female), race/ethnicity (non-Hispanic White or other). Model 2 was adjusted for age (continuous), sex (male or female), race/ethnicity, education level, family income-to-poverty ratio, previous drinking and smoking history, and presence of diabetes, hypertension, dyslipidemia, and current psoriatic severity, where appropriate.

**Figure 2:**
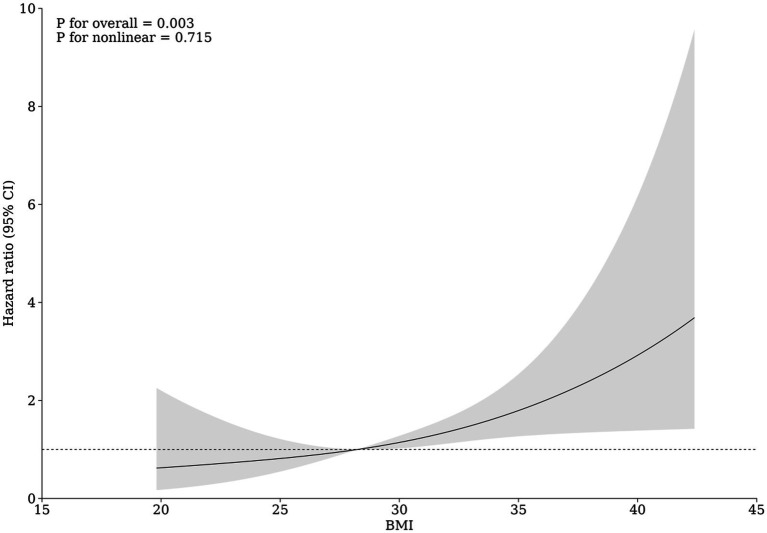
Association between BMI and the risk of all-cause mortality among psoriatic individuals.

Figure 2, via an RCS regression model, suggests an overall trend of linear relationship between BMI value and the risk of all-cause mortality among individuals with a history of psoriasis (p for nonlinearity = 0.715). According to the RCS model, when a BMI of 28.1 served as a reference for an HR of 1, the overall association was statistically significant (*p* = 0.003). On one hand, we found that that individuals with baseline BMI values below 28.1 tended to exhibit a slight reduction in HR (i.e., less than 1). Nonetheless, this trend lacks statistical significance. On the other hand, when BMI exceeds 28.1, there was a potentially linear significant correlation between BMI and the risk of all-cause mortality in this population.

### 3.5 Association between BMI and the current severity of psoriasis among participants with a history of psoriasis

Supplementary Table S6 indicates that an elevated BMI in psoriatic individuals was correlated with an increased probability of having severe psoriasis (HR = 1.057, 95% CI 1.003–1.113, *p* = 0.038). Also, those who had a BMI ≥30 were 6.369 times more likely to have more severe psoriasis compared to those with a BMI <30.

### 3.6 Mortality rate differences between individuals with mild psoriasis and those with moderate-to-severe psoriasis, based on BMI category

Supplementary Table S7 shows significant mortality rate differences between overweight individuals with mild psoriasis and obese individuals with moderate-to-severe (MtS) psoriasis (*p* = 0.032), as well as between lean individuals with mild psoriasis and obese individuals with MtS psoriasis (*p* = 0.045). However, we did not find such difference between lean individuals with mild psoriasis and overweight individuals with moderate-to-severe psoriasis (*p* = 0.371).

### 3.7 Sensitivity analyses

In our sensitivity analyses, the positive association between higher BMI and increased all-cause mortality risk among individuals with psoriasis was not materially altered when excluding participants who died within the first two years of follow-up (adjusted HR = 1.082, 95% CI 1.045–1.228, *p* < 0.001). Similar results were observed with the use of propensity score matching (PSM) (adjusted HR = 1.040, 95% CI 1.010–1.070, *p* = 0.004). The results remained substantially unchanged even after combining both criteria (adjusted HR = 1.050, 95% CI 1.020–1.080, *p* = 0.002). Refer to Supplementary Table S8.

The sensitivity analyses, conditioned on balancing the baseline variables, confirmed the robustness of the association between higher BMI and increased all-cause mortality in individuals with psoriasis in our study.

## 4 Discussion

To the best of our knowledge, the present study was the first to explore the relationship between BMI and the risk of all-cause mortality among individuals with psoriasis. More specifically, we analyzed data from the NHANES database collected between 2003 and 2006, as well as between 2009 and 2014, encompassing a total of 22,876 individuals aged 20 years and above, with or without a history of psoriasis. Our primary finding suggested a positive correlation between BMI and the risk of all-cause mortality among individuals (≥20) with a history of psoriasis. After adjusting for multiple potential confounders, BMI was found to independently increase all-cause mortality risk in this population, showing a trend where mortality risk increased by 5.5% for each unit rise in BMI. These results remained consistent even when utilizing a propensity score matching (PSM) approach. However, similar associations were not observed among individuals without histories of psoriasis in our study.

Despite these novel findings, our results from the restricted cubic splines (RCS) analysis and Multivariate Cox Regression model revealed nuanced results within the population of individuals with psoriasis. The RCS analysis indicated a potential dose–response relationship between baseline BMI and the risk of all-cause mortality. For BMIs ranging from 20 to 28.1, no significant correlation with mortality risk was identified, partially aligning with the results of our stratified analysis when considering BMI as a categorical variable. This stratified analysis suggested no difference in mortality risk between the lean and overweight groups. However, notably, from a BMI of 28.1 onwards, an evident increase in mortality risk was observed in the RCS analysis as BMI values climbed, suggesting a potential linear relationship within this higher BMI spectrum. By contrast, the Multivariate Cox Regression analysis indicated a continuous elevation in mortality risk, with a hazard ratio (HR) of 1.055 per unit increase in BMI across the entire BMI spectrum. This discrepancy between analyses may be attributed to methodological disparities. Compared to the RCS approach, which allowed for exploring such relationship across distinct BMI segments and offered insights into the behavior of this association at a more granular level, the Cox regression model provided an average effect size across the entire BMI range. This approach potentially overshadows subtleties within individual segments. Therefore, although we suggested a positive association between BMI and all-cause mortality risk, evidenced by significant results even after propensity score matching, the interaction between a BMI below 28.1 and mortality risk may be more complex than initially indicated. Further evidence is needed to investigate the impact of a BMI below the obesity threshold on mortality risk. Furthermore, where possible, future prospective studies are encouraged to include larger populations with psoriasis and conduct extended follow-ups to document dynamic changes in BMI, thereby providing comprehensive evidence to better explain and understand the current findings.

Although the impact of overweight on mortality risk is still debated, factors such as a BMI exceeding 30 (i.e., obesity), cigarette smoking, and being male consistently emerge as risk factors for higher all-cause mortality, irrespective of participants having psoriasis or not (16, 30–32). These associations persist in our study even after adjusting for multiple potential confounding covariates, including race/ethnicity, education level, family income-to-poverty ratio, and comorbidities, highlighting the protective effects of being non-obese, non-smoking, and female. Furthermore, in line with previous studies (30, 33), our results also indicated that overweight in older adults aged over 60 was not associated with increased mortality compared to a BMI < 25 group. This finding could be attributed to many elderly individuals having a relatively lower BMI due to unintentional weight loss from illness, potentially exacerbating illness due to sarcopenia (34). Conversely, higher weight increases the risk of developing multiple conditions such as diabetes, arthritis, fatty liver, hypertension, heart failure, and kidney disease (17).

Excessive adiposity (body fatness), a well-established risk factor for premature mortality, is primarily linked to various chronic diseases (14, 16). However, excess weight reflected by a higher BMI could not directly reflect excess body fat. It was suggested that BMI is a relatively poor indicator of body fat percentage and does not account for fat mass distribution across different body sites (35). The observation that there was no change in mortality within the BMI range of 22.5 to 30 in a previous study and below 28.1 in our study may be due to the weak association between BMI and adiposity within this range (15, 35). For example, prior research has shown that among individuals with a BMI of 25 kg/m^2^, body fat percentage in men ranged from 14 to 35%, and in women, it ranged from 26 to 43% (35), suggesting the instability of utilizing BMI to reflect one’s body fat.

Previous studies suggested an indirect correlation between BMI and mortality among non-obese individuals with psoriasis, indicating that a higher BMI may lead to a higher risk of severe psoriasis and subsequently to a higher risk of all-cause mortality (20, 21). In our study, we did observe a notably positive correlation between BMI and the risk of more severe psoriasis in non-obese group. However, we did not find a significant difference in mortality rate between lean individuals with mild psoriasis and overweight individuals with moderate-to-severe psoriasis. These results may indicate that the relationship between BMI and mortality between lean and overweight groups of individuals with psoriasis is far more complicated than what could be inferred indirectly based on prior findings (20, 21). For future studies, exploring alternative metrics to assess body fat mass and their impacts on these associations is encouraged, especially between lean weight and overweight groups. Moreover, in clinical practice, it is advisable to comprehensively consider the specific condition of a person with psoriasis when assessing their all-cause mortality risk based on BMI.

Overall, our study has several strengths. Firstly, the nationally representative sample size is commendable. Secondly, we adjusted for multiple potential confounding factors using the propensity score matching (PSM). Before PSM, the overweight group had the highest percentages of male participants, non-Hispanic Whites, and individuals with the highest Poverty Income Ratio (PIR), with significant differences noted. The post-PSM comparison increased the validity of our analysis by compensating for these baseline differences, thus ensuring a more balanced and unbiased assessment across the groups. Interestingly, the results remained consistent with those obtained before PSM, enhancing the reliability and stability of our findings. Thirdly, while prior findings indirectly suggest a positive association between BMI and all-cause mortality in non-obese psoriatic individuals, our study provided direct evidence that did not support this relationship. Lastly, our study contributed additional evidence regarding the insignificant association between BMI and all-cause mortality among the general population, consistent with findings from previous studies.

However, there are several limitations to our study. First, we relied on single baseline measures of BMI, precluding evaluation of the time-varying association of interest. Second, the questionnaire format of the psoriasis profile may introduce recall bias. Third, our results are based solely on U.S. adults, limiting generalizability to other populations. Finally, due to the limitations of using BMI to reflect body fat mass and the possibility of residual and unknown confounding factors, our results should be interpreted with caution.

## 5 Conclusion

In a nationally representative sample of U.S. adults, we observed a trend wherein a higher BMI was linked to an increased risk of all-cause mortality among individuals with psoriasis, but not among those without the condition. However, the definitive impact of overweight compared to normal weight or underweight remains inadequately explored. Further studies are needed to provide additional evidence on these relationships. Additionally, exploring alternative metrics for assessing body fat to investigate these associations is also urgent.

## Data availability statement

The raw data supporting the conclusions of this article will be made available by the authors, without undue reservation.

## Ethics statement

The studies involving humans were approved by National Health and Nutrition Examination Survey. The studies were conducted in accordance with the local legislation and institutional requirements. The participants provided their written informed consent to participate in this study.

## Author contributions

ZW: Writing – original draft, Conceptualization, Data curation, Formal analysis, Methodology. GN: Writing – original draft, Conceptualization, Data curation, Investigation, Methodology. CS: Writing – review & editing, Conceptualization, Formal analysis, Methodology. DS: Writing – review & editing, Conceptualization, Investigation, Methodology, Project administration, Resources, Supervision, Visualization, Writing – original draft.

## Footnotes

^1^ https://www.cdc.gov/nchs/nhanes/index.htm
