# Supplementary material for: Associations between body mass index and all-cause mortality among individuals with psoriasis: results from the NHANES database retrospective cohort study
Source: Front Nutr. 2024 Jun 5;11:1407454. doi: 10.3389/fnut.2024.1407454 (PMC11188368; doi:10.3389/fnut.2024.1407454)
Supplement: Supplementary file 1 [file Data_Sheet_1.docx]

**Associations Between Body Mass Index and All-Cause Mortality Among Individuals with Psoriasis: Results from the NHANES database retrospective cohort study**

**Supplementary Materials**

**Contents**

[**Figure S1. Kaplan–Meier survival analysis among individuals with psoriasis.** 2](#_Toc161412989)

[**Table S1. Baseline characteristics among all participants.** 3](#_Toc161412990)

[**Table S2. Baseline characteristics of psoriatic individuals based on obese status after Propensity Score-Matching (PSM).** 4](#_Toc161412991)

[**Table S3. Baseline characteristics of psoriatic individuals based on obese status before and after PSM combined with the exclusion of participants who died within the first two years of follow-up.** 5](#_Toc161412992)

[**Table S4. Mortality rate according to various causes of death, based on BMI category among individuals with psoriasis.** 6](#_Toc161412993)

[**Table S5. Multivariate Cox Regression for all-cause mortality in individuals without psoriasis.** 7](#_Toc161412994)

[**Table S6. Multivariate Cox Regression for severity of psoriasis in individuals with psoriasis.** 8](#_Toc161412995)

[**Table S7. Fisher's exact test for mortality rate differences between individuals with mild psoriasis and those with moderate-to-severe psoriasis, based on BMI category.** 9](#_Toc161412996)

[**Table S8 Sensitivity analyses among psoriatic individuals.** 10](#_Toc161412997)

**Figure S1. Kaplan–Meier survival analysis among individuals with psoriasis.**


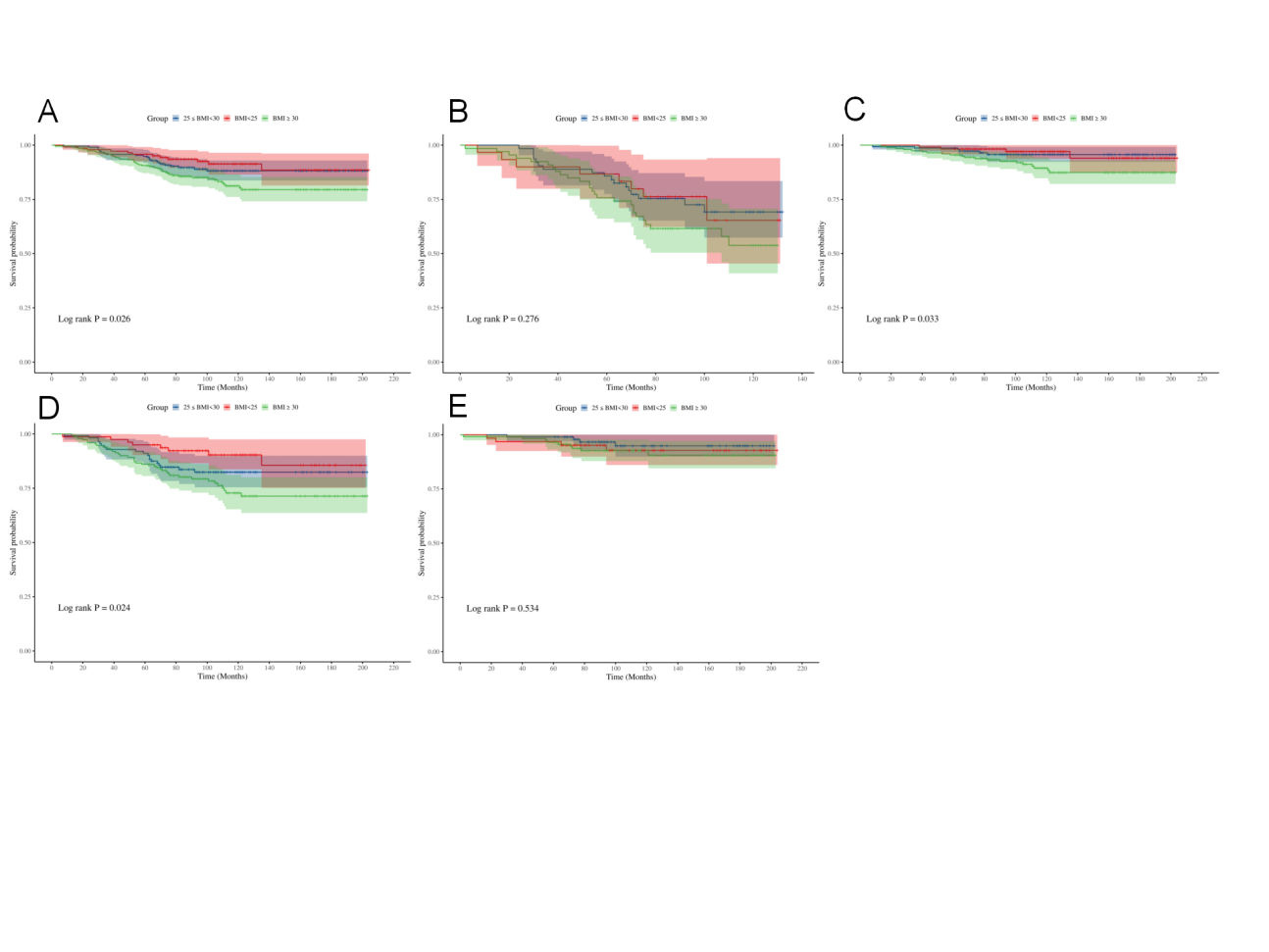


A. Kaplan–Meier survival analysis of all individuals with psoriasis; B. Kaplan–Meier survival analysis of psoriatic individuals age>60; C. Kaplan–Meier survival analysis of psoriatic individuals age≤60; D. Kaplan–Meier survival analysis of psoriatic smokers; E. Kaplan–Meier survival analysis of psoriatic non-smokers.

**Table S1. Baseline characteristics among all participants.**

|  | **Without psoriasis** | **With psoriasis** | ***p* Value** |
| --- | --- | --- | --- |
|  | **(n=22258)** | **(n=618)** |  |
| **Age, years old** | 45.84±16.98 | 49.31±16.25 | <0.001 |
| **Male, n%** | 10695(48.05) | 289(46.76) | 0.528 |
| **Race/Ethnicity** |  |  | <0.001 |
| Mexican American | 3598(16.17) | 51(8.25) |  |
| Other Hispanic | 1815(8.15) | 48(7.77) |  |
| Non-Hispanic White | 9660(43.40) | 375(60.68) |  |
| Non-Hispanic Black | 4945(22.22) | 83(13.43) |  |
| Other Race | 2240(10.06) | 51(9.87) |  |
| **Educational attainment** |  |  | 0.103 |
| Less than 9th grade | 2157(9.69) | 41(6.63) |  |
| 9-11th grade | 3300(14.83) | 82(13.27) |  |
| High school | 5042(22.65) | 139(22.49) |  |
| Some college or AA degree | 6700(30.10) | 198(32.04) |  |
| College graduate or above | 5059(22.73) | 158(25.57) |  |
| **PIR** | 2.52±1.65 | 2.60±1.70 | 0.282 |
| **BMI, kg/m^2^** | 28.91±6.94 | 29.18±5.78 | 0.589 |
| **Hypertension** | 4441(19.95) | 136(22.01) | 0.569 |
| **Diabetes** | 1496(6.72) | 40(6.47) | 0.753 |
| **Dyslipidemia** | 3875(17.41) | 131(21.20) | 0.125 |
| **Smoking** | 9817(44.11) | 344(55.66) | <0.001 |
| **Drinking** | 7695(34.57) | 219(35.44) | 0.289 |

Abbreviation: AA, the associate in arts degree. PIR, poverty income ratio.

**Table S2. Baseline characteristics of psoriatic individuals based on obese status after Propensity Score-Matching (PSM).**

|  | **Non-obese** | **Obese** | ***p* Value** |
| --- | --- | --- | --- |
|  | (n=326) | (n=241) |  |
| Age, years old | 49.22 ± 17.04 | 49.94 ± 15.10 | 0.595 |
| Male, n% | 161 (49.39) | 107 (44.40) | 0.240 |
| Race/Ethnicity |  |  | 0.181 |
| Mexican American | 18(5.52) | 16(6.64) |  |
| Other Hispanic | 24(7.36) | 21(8.71) |  |
| Non-Hispanic White | 221(67.79) | 148 (61.41) |  |
| Non-Hispanic Black | 38(11.66) | 43(17.84) |  |
| Other Race | 25(7.67) | 13(5.39) |  |
| Educational attainment |  |  | 0.124 |
| Less than 9th grade | 20(6.13) | 14(5.81) |  |
| 9-11th grade | 47(14.42) | 31(12.86) |  |
| High school | 72 (22.09) | 57(23.65) |  |
| Some college or AA degree | 94(28.83) | 90(37.34) |  |
| College graduate or above | 93(28.53) | 49(20.33) |  |
| PIR | 2.69 ± 1.69 | 2.47 ± 1.62 | 0.117 |
| Hypertension | 69(21.17) | 50(20.75) | 0.787 |
| Diabetes | 22(6.75) | 13(5.39) | 0.860 |
| Dyslipidemia | 70(21.47) | 46(19.09) |  |
| Smoking | 185(56.75) | 138(57.26) | 0.903 |
| Drinking | 101(30.98) | 93(38.59) | 0.056 |
| Severity of psoriasis |  |  | 0.908 |
| Mildly | 267(81.90) | 193(80.12) |  |
| Moderate-to-severe | 59(18.10) | 48(19.88) |  |

Abbreviation: AA, the associate in arts degree. PIR, poverty income ratio.

**Table S3. Baseline characteristics of psoriatic individuals based on obese status before and after PSM combined with the exclusion of participants who died within the first two years of follow-up.**

|  | **Before PSM** | | |  | **After PSM** | |  |
| --- | --- | --- | --- | --- | --- | --- | --- |
|  |  |  |  |  |  |  |  |
|  | non-Obese | Obese | p value |  | non-Obese | Obese | p value |
|  | (n=348) | (n=258) |  |  | (n=318) | (n=234) |  |
| Age, years old | 48.62 ± 16.87 | 49.39 ± 15.08 | 0.555 |  | 48.66 ± 16.81 | 49.29 ± 14.99 | 0.647 |
| Male, n% | 172 (49.43) | 108 (41.86) | 0.065 |  | 160 (50.31) | 102 (43.59) | 0.118 |
| Race/Ethnicity |  |  | 0.001 |  |  |  | 0.366 |
| Mexican American | 18 (5.17) | 33 (12.79) |  |  | 18 (5.66) | 17 (7.26) |  |
| Other Hispanic | 25 (7.18) | 22 (8.53) |  |  | 23 (7.23) | 20 (8.55) |  |
| Non-Hispanic White | 221 (63.51) | 146 (56.59) |  |  | 216 (67.92) | 145 (61.97) |  |
| Non-Hispanic Black | 38 (10.92) | 44 (17.05) |  |  | 38 (11.95) | 39 (16.67) |  |
| Other Race | 46 (13.22) | 13 (5.04) |  |  | 23 (7.23) | 13 (5.56) |  |
| Educational attainment |  |  | 0.052 |  |  |  | 0.077 |
| Less Than 9th Grade | 22 (6.32) | 18 (6.98) |  |  | 19 (5.97) | 13 (5.56) |  |
| 9-11th Grade | 47 (13.51) | 34 (13.18) |  |  | 45 (14.15) | 29 (12.39) |  |
| High School | 77 (22.13) | 60 (23.26) |  |  | 72 (22.64) | 55 (23.50) |  |
| Some College or AA degree | 97 (27.87) | 94 (36.43) |  |  | 88 (27.67) | 88 (37.61) |  |
| College Graduate or above | 105 (30.17) | 52 (20.16) |  |  | 94 (29.56) | 49 (20.94) |  |
| PIR | 2.73 ± 1.67 | 2.44 ± 1.61 |  |  | 2.71 ± 1.68 | 2.52 ± 1.62 | 0.194 |
| Hypertension | 76(21.84) | 56(21.71) | 0.037 |  | 67(21.07) | 50(21.37) | 0.922 |
| Diabetes | 25(7.18) | 15(5.81) | 0.850 |  | 22(6.92) | 13(5.56) | 0.847 |
| Dyslipidemia | 78(22.41) | 51(19.77) | 0.908 |  | 69(21.70) | 45(19.23) | 0.757 |
| Smoking | 190 (54.60) | 145 (56.20) | 0.695 |  | 180 (56.60) | 134 (57.26) | 0.877 |
| Drinking | 113(32.47) | 102(39.53) | 0.067 |  | 94(29.56) | 87(37.18) | 0.076 |
| Severity of psoriasis |  |  | 0.685 |  |  |  | 0.876 |
| mildly | 285(81.89) | 210(81.22) |  |  | 276(81.90) | 187(79.99) |  |
| moderate-to-severe | 63 (18.11) | 48 (18.78) |  |  | 42 (18.10) | 47(20.01) |  |

**Table S4. Mortality rate according to various causes of death, based on BMI category among individuals with psoriasis.**

|  | BMI<25  (n=142) | 25≤BMI<30  (n=211) | BMI≥30  (n=265) | Adjusted *p-value* |
| --- | --- | --- | --- | --- |
| No. of subjects |  |  |  |  |
| CVD-related mortality | 3(19%) | 3(19%) | 10(63%) | 0.005 |
| Malignant neoplasms | 3(11%) | 10(36%) | 15(54%) | 0.887 |
| Chronic lower respiratory  diseases | 0 | 3(75%) | 1(25%) |  |
| Accidents | 1(33%) | 1(33%) | 1(33%) |  |
| Cerebrovascular diseases | 0 | 1(33%) | 2(67%) |  |
| Alzheimer’s disease | 0 | 0 | 0 |  |
| Diabetes | 0 | 1(25%) | 3(75%) |  |
| Influenza and pneumonia | 2(100%) | 0 | 0 |  |
| Nephritis, nephrotic  syndrome and nephrosis | 0 | 0 | 3(100%) |  |
| All other causes | 3(17%) | 4(22%) | 11(61%) | 0.009 |

All p values were adjusted for age, sex, race/ethnicity, education level, family income-to-poverty ratio, previous drinking and smoking histories, and current psoriatic severity, where appropriate.

**Table S5. Multivariate Cox Regression for all-cause mortality in individuals without psoriasis.**

|  |  | HR | 95% CI | p Value |
| --- | --- | --- | --- | --- |
| BMI |  |  |  |  |
|  | Crude | 1.001 | 0.994-1.007 | 0.822 |
|  | Model 1 | 1.003 | 0.996-1.010 | 0.390 |
|  | Model 2 | 0.996 | 0.964-1.008 | 0.553 |
|  |  |  |  |  |
| BMI<25 |  |  |  |  |
|  | Crude | 0.985 | 0.896-1.082 | 0.748 |
|  | Model 1 | 1.231 | 1.116-1.358 | <0.001 |
|  | Model 2 | 1.341 | 1.150-1.563 | <0.001 |
|  |  |  |  |  |
| 25≤BMI<30 |  |  |  |  |
|  | Crude | 0.973 | 0.886-1.069 | 0.571 |
|  | Model 1 | 0.807 | 0.731-0.890 | <0.001 |
|  | Model 2 | 0.847 | 0.726-0.988 | 0.034 |
|  |  |  |  |  |
| BMI≥30 |  |  |  |  |
|  | Crude | 1.041 | 0.951-1.139 | 0.386 |
|  | Model 1 | 1.021 | 0.929-1.123 | 0.667 |
|  | Model 2 | 0.905 | 0.781-1.050 | 0.189 |

**Table S6. Multivariate Cox Regression for severity of psoriasis in individuals with psoriasis.**

|  |  | HR | 95% CI | p Value |
| --- | --- | --- | --- | --- |
| BMI |  |  |  |  |
|  | Crude | 1.046 | 1.011-1.081 | 0.009 |
|  | Model 1 | 1.054 | 1.019-1.091 | 0.002 |
|  | Model 2 | 1.057 | 1.003-1.113 | 0.038 |
| BMI≥30 |  |  |  |  |
| vs. BMI <30 | Crude | 2.139 | 1.178-3.884 | 0.012 |
|  | Model 1 | 2.611 | 1.423-4.793 | 0.002 |
|  | Model 2 | 6.369 | 2.037-19.915 | 0.001 |

**Table S7. Fisher's exact test for mortality rate differences between individuals with mild psoriasis and those with moderate-to-severe psoriasis, based on BMI category.**

| **Group** | **Fisher’s exact test p-value** |
| --- | --- |
| Mild Psoriasis (Lean) | *Reference* |
| MtS Psoriasis (Overweight) | 0.371 |
| Mild Psoriasis (Overweight) | *Reference* |
| MtS Psoriasis (Obese) | 0.032 |
| Mild Psoriasis (Lean) | *Reference* |
| MtS Psoriasis (Obese) | 0.045 |

Abbreviation: MtS, Moderate-to-severe.

**Table S8 Sensitivity analyses among psoriatic individuals.**

|  |  | **Adjusted HR** | **95% CI** | ***p Value*** |
| --- | --- | --- | --- | --- |
| **(1). Excluding participants who died in the first two years of follow-up** | | 1.082 | 1.045-1.228 | <0.001 |
| **(2). After propensity score matching** | | 1.040 | 1.010-1.070 | 0.004 |
| **(3). After propensity score matching as well as excluding participants who died in the first two years of follow-up** | | 1.050 | 1.020-1.080 | 0.002 |
